# Comparison of CTS5 risk model and 21-gene recurrence score assay in large-scale breast cancer population and combination of CTS5 and recurrence score to develop a novel nomogram for prognosis prediction

**DOI:** 10.1016/j.breast.2022.03.007

**Published:** 2022-03-16

**Authors:** Changjun Wang, Ying Xu, Yan Lin, Yidong Zhou, Feng Mao, Xiaohui Zhang, Songjie Shen, Yanna Zhang, Qiang Sun

**Affiliations:** Department of Breast Surgery, Peking Union Medical College Hospital, Chinese Academy of Medical Sciences and Peking Union Medical College, Beijing, 100730, China

**Keywords:** Breast cancer, Oncotype, Recurrence score, Clinical treatment score post–5 years, Clinical risk model, BC, Breast cancer, CTS5, Clinical treatment score post–5 years, ATAC trial, Arimidex, Tamoxifen, alone or in combination trial, BIG, Breast International group, HR, Hormone receptor, HER2, Human epidermal growth factor receptor-2, RS, Recurrence score, NSABP, National surgical adjutant breast project, DFS, Disease-free survival, OS, Overall survival, TAILORx, Trial assigning Individualized options for treatment, SEER database, Surveillance, epidemiology, and end results program, BCSS, Breast cancer specific survival, ROC curve, Survival receiver operating characteristic curve, NRI, Net reclassification index, CI, Confidence interval: Hazard ratio, AUC, Area under curve, LN, lymph node, DCA, Decision curve analysis

## Abstract

**Background:**

Breast cancer is the most common malignancy in women. Clinical models such as Oncotype DX recurrence score (RS) and Clinical Treatment Score post–5 years (CTS5) model for survival prediction are crucial for clinical practice. However, it remains unclear whether CTS5 or RS would be a more powerful clinical model for recurrence risk evaluation. Therefore, we conducted the present study to compare the performance of CTS5 risk model and RS on different recurrence evaluation. And we further integrated the two models into a novel nomogram to improve the power for prognosis prediction.

**Methods:**

Female patients with invasive hormone receptor positive breast cancer in the Surveillance, Epidemiology, and End Results Program (SEER) database with RS data available were included. The clinicopathological data were directly extracted from SEER database. Participants were divided into three subsets according to recurrence timing (<36 months, between 36 and 60 months, and >60 months) for model evaluation**.** Survival receiver operating characteristic curve and C-index were calculated to evaluate discrimination. Calibration curve were used to visual inspection for calibration. Model comparison was assessed by net reclassification index (NRI) method. Nomogram prognostic model was developed with the combination of CTS5 score and RS and also included other critical clinicopathological parameters.

**Results:**

In total, 64044 breast cancer patients were enrolled for analysis. The number of patients with survival <36 months (early recurrence subset), 36–60 months (intermediate recurrence subset) and >60 months (late recurrence subset) were 64044, 36878 and 15926, respectively. For model discrimination, CTS5 model was superior to RS for overall survival (OS) prediction (likelihood ratio test *P* < 0 0.001). RS model showed better performance for breast cancer specific survival (BCSS) in late recurrence subsets and worse performance in early and intermediate recurrence subsets than CTS5 (likelihood ratio test *P* < 0 0.001). For calibration, CTS5 model was superior to RS model for OS, which overestimated the recurrence risk in low-risk subgroup. Both models overestimated the risk for BCSS. In either early/intermediate/late recurrence patient subsets, there was no significant difference in NRI between two models in terms of both BCSS and OS, indicating the two models had comparable prognostic value. The nomogram which combined these two models largely improved the discrimination and calibration power (C-index 0.70–0.72).

**Conclusions:**

Our study proved the CTS5 risk model had comparable prognostic value as RS in HR + breast cancer patients. And the novel nomogram model had better discrimination and calibration than both CTS5 and RS, and future large-scale clinical trials are warranted for further validation.

## 1 Introduction

Breast cancer (BC) is the most common malignancy in women and accounts for 30% of female cancers [1]. Clinical models for predicting the short-term and long-term survival of breast cancer are crucial for clinical practice. Hence, Dowsett and his colleagues developed a simple model which was called “Clinical Treatment Score post–5 years (CTS5) risk model” to estimate risk of late distant recurrence [2]. This model was developed based on the data from the ATAC (Arimidex, Tamoxifen, Alone or in Combination) trial and the BIG (Breast International Group) 1–98 trial. It involved several key clinicopathologic factors, such as age, grade and tumor size, to estimate the late recurrence risk and divided the patients into three subgroups with different recurrence risk. CTS5 model had advantage that it was built based on the data from two large randomized clinical trial with 10 years survival information. However, it was criticized for no validation for human epidermal growth factor receptor-2 positive (HER2+) patients and no premenopausal patients included in the training dataset. And it may overestimate the recurrence rate for hormone receptor (HR) positive breast cancer [3]. Additionally, with the prevalence of multigene expression profiles, CTS5 model involved only clinicopathological parameters without taking genomic information into consideration. Therefore, the prognostic value of CTS5 model may be improved with the integration of genomic data and need to be further validated in large-scale population, and the correlation between model accuracy and recurrence timing courses also warrants further investigation.

The Oncotype DX is a widely used genomic assay that calculates recurrence score (RS) to facilitate clinical decision making for adjuvant chemotherapy [4]. National Surgical Adjutant Breast Project (NSABP)-B14 and B20 study validated RS as a continuous parameter could serve as a predictor for recurrence [4,5]. NSABP-B28 trial further proved that RS was an independent predictor for both disease-free survival (DFS) and overall survival (OS) [6]. Study by Paik et al. also suggested RS could provide long-term prognostic information up to 10-year survival [4]. Trial Assigning Individualized Options for Treatment (TAILORx) was one of the largest prospective studies to assess the clinical validity of RS and demonstrated patients with a favorable gene-expression profile had very low rates of recurrence at 5 years with endocrine therapy alone (5-year DFS 93.8%, OS 98%) [7].

However, several studies reported RS was less accurate for predicting late recurrence (over 5 years), and it remains unclear whether CTS5 or RS would be a more powerful clinical model for recurrence risk evaluation. Moreover, given RS provide additional information from the genomic perspective, the combination of CTS5 and RS may improve the performance of the whole risk prediction model. Therefore, we conducted the present study based on large-scale data from Surveillance, Epidemiology, and End Results Program (SEER) database to compare the performance of CTS5 risk model and RS on recurrence prediction, and investigate the correlation between model performance and recurrence timing course. And we further integrated the two models into a novel nomogram to improve the power for prognosis prediction.

## 2 Methods

### 2.1 Study population and data collection

SEER database is a population-based database founded by National Cancer Institute. It is an open-access resource for cancer-based epidemiology and survival analyses (See Website “https://seer.cancer.gov/data/” for detailed information). Data access for present study was authorized by SEER Program and relevant case lists were extracted from SEER 18 incidence database (released April 2016, based on the November 2015 submission). SEER*Stat software from the National Cancer Institute (Surveillance Research Program, National Cancer Institute SEER*Stat software, http://www.seer.cancer.gov/seerstat) (Version 8.3.5) was used to identify eligible patients. The data released by the SEER database do not require patient informed consent since cancer is a reportable disease in United States and the procedures are in accordance with the ethical standards of the institutional/national research committee and the 1964 Helsinki declaration with its later amendments or comparable ethical standards.

Female patients with invasive HR + breast cancer in the SEER database with RS data available were included. The clinicopathological data including patient characteristics, survival months, age, race, tumor size, histological grade, lymph node metastasis status, TNM Stage, HR status, HER2 status, treatment and RS were directly extracted from SEER database. Please see Fig. S1 for flowchart of patient selection. Participants were divided into three subsets according to survival time (<36 months, between 36 and 60 months, and >60 months) for model evaluation**.**

### 2.2 Statistical analysis

The CTS5 score was calculated using the following equation: CTS5 = 0.438*nodes +0.988*(0.093*size ^2^- 0.001*size^2^ + 0.375*grade + 0.017*age). And patients were further classified into low, intermediate and high-risk subgroups with CTS5 score <3.13 (recurrence risk <5%), 3.13 to 3.86 (recurrence risk 5%–10%) and >3.86 (recurrence risk >10%), respectively [2]. RS was extracted from SEER database and patients could also be classified into three different risk subgroups with RS 18 and 30 as cutoff points (low risk <18, intermediate risk 18–30 and high risk >30).

The primary endpoints were OS and breast cancer specific survival (BCSS). OS was calculated as the time period from the date of cancer diagnosis until the last date for which completed vital status data were available. BCSS was defined as the time between breast cancer diagnosis and death due to breast cancer Kaplan-Meier method were used for survival data analysis.

Discrimination and calibration were two key features for model performance [8]. Survival Receiver Operating Characteristic (ROC) curve and C-index were calculated to evaluate discrimination [9]. Calibration curve were used for visual inspection of calibration [10]. Model comparison was assessed by net reclassification index (NRI) method [8]. Multivariate Cox regression model and backward stepwise selection based on Bayesian Information Criterion was adopted to determine variables that involved in the model. Nomogram prognostic model was developed with the combination of CTS5 score and RS and also included other critical clinicopathological parameters. The nomogram was constructed for predicting 1-year, 3-year and 5-year OS and BCSS. Statistical analyses were performed using R (4.0.3) software. All the statistical tests were two-sided, and statistical significance was defined as *P* value < 0.05.

## 3 Results

### 3.1 Demographics and clinicopathological characteristics

In total, 64044 breast cancer patients were enrolled for analysis. The median follow-up was 41 (0–83) months. The number of patients with survival <36 months (early recurrence subset), 36–60 months (intermediate recurrence subset) and >60 months (late recurrence subset) were 64044, 36878 and 15926, respectively. The demographics and clinicopathological characteristics of the three subsets were comparable. Please see Table 1 for details.

**Table 1:** Demographic and Clinical Characteristics of included Patients.

Characteristic	Early recurrence subset N = 64044 (%)	Intermediate recurrence subset N = 36878（%）	Late recurrence subset N = 15926（%）	*P* value
Age				0.252
Mean ± sd	58.29 ± 10.55	58.07 ± 10.54	57.85 ± 10.45	
≤45	6494 (10.1)	3783 (10.3）	1641 (10.3）	
45–65	37992 (59.3)	22199 (60.2)	9775 (61.4)	
＞65	19558 (30.6)	10896 (29.5)	4510 (28.3)	
Race				<0.001
White	52290 (81.6)	30456 (82.6)	13333 (83.7)	
Black	5308 (8.3)	2957 (8.0)	1168 (7.3)	
Asian	5761 (9.0)	3121 (8.5)	1287 (8.1)	
Other	282 (0.4)	161 (0.4)	64 (0.4)	
Unknown	403 (0.6)	183 (0.5)	74 (0.5)	
Tumor size（mm）				0.491
≤20	46627 (72.8)	27177 (73.7)	11953 (75.1)	
20–50	16224 (25.3)	9055 (24.6）	3719 (23.3）	
＞50	1193 (1.9)	646 (1.8）	254 (1.6）	
Grade				0.099
I	18541 (29.0)	10683 (29.0)	4714 (29.6)	
II	35251 (55.0）	20199 (54.8）	8639 (54.2）	
III	10252 (16.0）	5996 (16.2）	2573 (16.2）	
LN（+）				0.618
0	53241 (83.1)	30974 (84.0)	13591 (85.3)	
1–3	10319 (16.1)	5644 (15.3)	2248 (14.1)	
4–9	375 (0.6)	209 (0.6)	69 (0.4)	
＞9	109 (0.2)	51 (0.1)	18 (0.1)	
ER				0.253
Negative	63 (0.1)	44 (0.1)	23 (0.1)	
Positive	63981 (99.9)	36834 (99.9)	15903 (99.9)	
PR				0.026
Negative	5380 (8.4)	3135 (8.5)	1444 (9.1)	
Positive	58664 (91.6）	33743 (91.5)	14482 (90.9)	
HER2				<0.001
Negative	62826 (98.1)	36080 (97.8)	15483 (97.2)	
Positive	1218 (1.9）	798 (2.2）	443 (2.8)	
Subtype				<0.001
Luminal A	62826 (98.1)	36080 (97.8)	15483 (97.2)	
Luminal B	1218 (1.9）	798 (2.2）	443 (2.8)	
TNM Stage				0.551
I	42854 (66.9)	25106 (68.1)	11100 (69.7)	
II	20176 (31.5）	11239 (30.6）	4626 (29.0）	
III	916 (1.4)	492 (1.3)	181 (1.1)	
IV	98 (0.2)	41 (0.1)	19 (0.1)	
Surgery				0.081
No	62 (0.1)	24 (0.07)	8 (0.05)	
Yes	63964 (99.87）	36843 (99.91)	15915 (99.93)	
Unknown	18 (0.03)	11 (0.03)	3 (0.02)	
Radiotherapy				0.271
No	23281 (36.4)	13659 (37.0)	5866 (36.8)	
Yes	39228 (61.3)	22519 (61.1)	9847 (61.8)	
Unknown	1535 (2.4)	700 (1.9)	213 (1.3)	
Chemotherapy				<0.001
No	50668 (79.1)	28804 (78.1)	12302 (77.2)	
Yes	13376 (20.9)	8074 (21.9)	3624 (22.8)	
RS risk				0.329
low	37897 (59.2)	21500 (58.3)	9002 (56.5)	
Intermediate	21750 (33.9)	12837 (34.8)	5769 (36.2)	
High	4397 (6.9)	2541 (6.9)	1155 (7.3)	
CTS5 risk				0.329
low	40084 (62.6)	23398 (63.4)	10387 (65.2)	
Intermediate	17881 (27.9)	10211 (27.7)	4329 (27.2)	
High	6079 (9.5)	3269 (8.9)	1210 (7.6)	

### 3.2 Discrimination

**Fig. 1:**
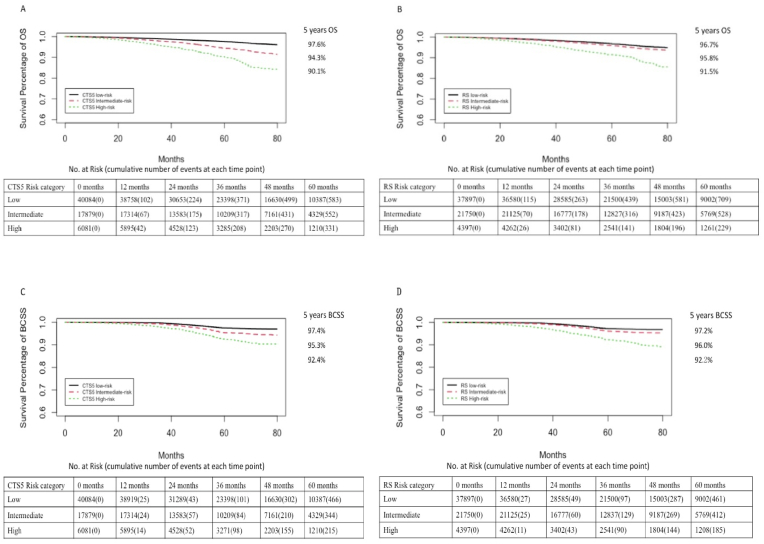
(A) (B) Kaplan-Meier survival estimates of observed OS rates with accompanying risk table, indicating discriminatory prognostic value of CTS5 and RS, respectively. (C) (D) Kaplan-Meier survival estimates of observed BCSS rates with accompanying risk table, indicating discriminatory prognostic value of CTS5 and RS.

In the CTS5 risk model, the 5 years OS rate and BCSS rate were 97.6% (95% confidence interval [CI], 97.3%–97.8%) and 97.4% (95% CI, 97.2%–97.7%) in the low-risk group, 94.3% (95% CI 93.8%–94.8%) and 95.3% (95% CI 94.8%–95.8%) in the intermediate-risk group, and 90.1% (95% CI, 89.0%–91.2%) and 92.4% (95% CI, 91.3%–93.5%) in the high-risk group, respectively. The OS and BCSS were significantly lower in the high-risk group (OS: hazard ratio [HR], 4.13; 95% CI, 3.64–4.68; BCSS: HR, 3.47; 95% CI, 2.97–4.05; log-rank P < 0 0.001) and the intermediate-risk group (OS:HR, 2.12; 95% CI, 1.91–2.37; BCSS: HR, 1.87; 95% CI, 1.64–2.13; log-rank P < 0.001) compared with the low-risk group (Fig. 1A and C).

For RS, the 5 years OS and BCSS rate were 96.7% (95% CI, 96.4%–96.9%) and 97.2% (95% CI, 96.9%–97.4%) in the low-risk group, 95.8% (95% CI 95.4%–96.2%) and 96.0% (95% CI 95.6%–96.4%) in the intermediate-risk group, and 91.5% (95% CI, 90.3%–92.6%) and 92.2% (95% CI, 90.9%–93.2%) in the high-risk group, respectively. The OS and BCSS were significantly lower in the high-risk group (OS:HR, 2.77; 95% CI, 2.42–3.18; BCSS: HR, 3.53; 95% CI, 2.99–4.16; log-rank *P* < 0 0.001) and the intermediate-risk group (OS:HR, 1.25; 95% CI, 1.13–1.39; BCSS: HR, 1.52; 95% CI, 1.34–1.73; log-rank *P* < 0.001) compared with the low-risk group (Fig. 1B, D).

Then patients included were divided into early/intermediate/late recurrence subsets. The corresponding survival curves for all the subsets were shown in Fig. S2 and Fig. S3. The BCSS were significantly lower in the high-risk group (log-rank P < 0 0.001) and the intermediate-risk group (log-rank *P* < 0.001) compared with the low-risk group in all three subsets for both two models. RS cannot clearly distinguish low-risk from intermediate-risk group in terms of OS [ HR 1.22 (95%CI 0.92–1.62), *P* = 0.164] in late recurrence subset. For early and intermediate recurrence subset, both CTS5 and RS showed good discriminating power for OS. In early recurrence subset, HR of intermediate-risk and high-risk groups were 1.94 (95%CI 1.67–2.67) and 3.89 (95%CI 3.28–4.62) respectively according to CTS5 model and 1.22 (95%CI 1.05–1.41) and 2.72 (95%CI 2.25–3.30) respectively according to RS model. In intermediate recurrence subset, HR of intermediate-risk and high-risk groups were 2.51 (95%CI 2.10–3.01) and 4.22 (95%CI 3.39–5.25) respectively according to CTS5 model and 1.30 (95%CI 1.09–1.54) and 2.69 (95%CI 2.13–3.40) respectively according to RS model. Details of HR and 95% CI were summarized in Table S1.

**Fig. 2:**
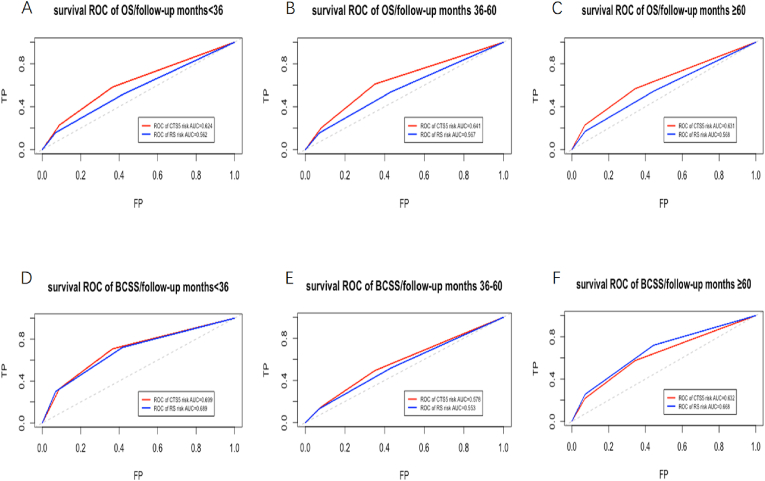
Survival ROC of OS (5A-5C) and BCSS (5D-F) according to CTS5 risk model and RS risk model for patients with different follow-up months of three subgroups.

Both the ROC curve and the C statistic that represented the probability that risk prediction is higher for a random patient with an event than without an event [8]. The survival ROC curve of OS and BCSS for two models were shown in Fig. 2. Details of Area Under Curve (AUC) and C-index were summarized in Table 2. The AUC of early/intermediate/late recurrence subsets of OS were 0.624, 0.641 and 0.631 for CTS5 model, while for RS were 0.562, 0.567 and 0.568. The AUC of early/intermediate/late recurrence subsets of BCSS were 0.699, 0.587 and 0.634 for CTS5 model, while for RS were 0.689, 0.553 and 0.669. CTS5 model was superior to RS in discrimination for OS (likelihood ratio test *P* < 0 0.001). RS model showed better performance in prediction of BCSS in late recurrence subsets and worse performance in early and intermediate recurrence subsets (likelihood ratio test *P* < 0 0.001).

**Table 2:** C-index and 95%CI of OS and BCSS according to CTS5 risk model and RS risk model.

Subset	Survival Rate	Model	Survival AUC	C-index (95%CI)	P (Likelihood-ratio test)
Early Recurrence subset	OS				<0.001
		CTS5 risk	0.624	0.622 (0.603–0.640)	
		RS risk	0.562	0.560 (0.542–0.579)	
	BCSS				<0.001
		CTS5 risk	0.699	0.695 (0.665–0.726)	
		RS risk	0.689	0.682 (0.650–0.714)	
Intermediate recurrence subset	OS				<0.001
		CTS5 risk	0.641	0.636 (0.615–0.658)	
		RS risk	0.567	0.570 (0.547–0.592)	
	BCSS				<0.001
		CTS5 risk	0.578	0.573 (0.555–0.593)	
		RS risk	0.553	0.557 (0.551–0.571)	
Late recurrence subset	OS				<0.001
		CTS5 risk	0.631	0.636 (0.598–0.673)	
		RS risk	0.568	0.561 (0.524–0.597)	
	BCSS				<0.001
		CTS5 risk	0.634	0.632 (0.569–0.699)	
		RS risk	0.669	0.668 (0.594–0.715)	

### 3.3 Calibration

**Fig. 3:**
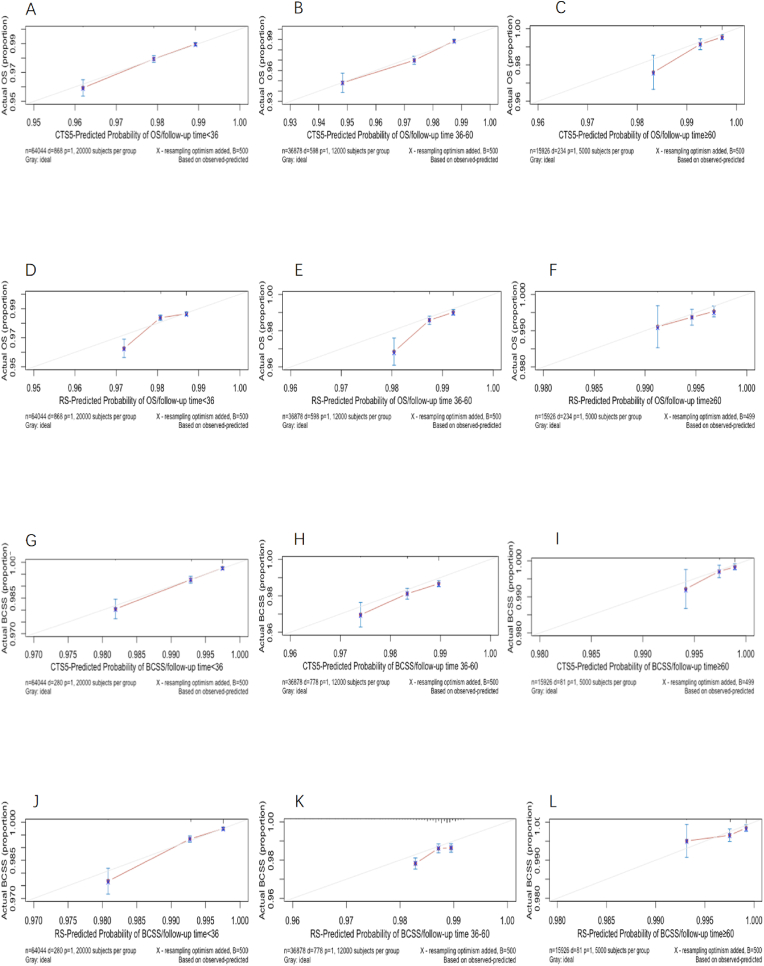
Calibration curve of OS and BCSS according to CTS5 risk model and RS risk model for patients with different follow-up months.

The calibration curve of two model demonstrated a comparable calibration ability for all the different risk subgroups (Fig. 3). Both two models revealed high agreement between prediction and observation probability for all subsets in terms of both BCSS and OS. For OS, the calibration of CTS5 model was superior to RS model which overestimated the risk in low-risk subgroup (Fig. 3A–F). Both models overestimated the risk of BCSS (Fig. 3G-L).

The deviation between prediction and observation had a negative correlation with absolute survival rate of OS and BCSS. The larger the OS and BCSS rates were, the less the overestimation of risk was.

### 3.4 Comparison of CTS5 model and RS model

NRI was adopted for model comparison. CTS5 model was set as control, and based on the classification of CTS5, NRI evaluated the proportions of patients who changed the class when RS model was added as a second classification tool. In another word, NRI reflected whether a new model could assign more patients into the correct classes. In either early/intermediate/late recurrence patient subsets, there was no significant difference in NRIs between two models in terms of both BCSS and OS, indicating the two models had comparable prognostic value. The NRI of early/intermediate/late recurrence subsets were −0.03(95%CI -0.036-0.319), −0.182(95%CI -0.253-0.115) and −0.181 (95%CI -0.332-0.632). Survival NRIs OS and BCSS were summarized in Tables S2 and S3.

### 3.5 Nomogram development with combination of CTS5 and RS

Given CTS5 and RS used clinicopathological and genomic information without significant overlap, the combination of these two models would potentially improve model performance. Hence, the present study included CTS5, RS and other clinically relevant parameters to build up a novel nomogram to facilitate clinical decision making. For nomogram construction and validation, three-fourth of all patients were set as the training set (n = 48033) and one-fourth as the validation set (n = 16011). The demographics and clinicopathological characteristics of the two subsets were summarized in Table S4. Variables selection was performed by the backward stepwise method (*P*＜0.05). Finally, Age, Tumor size, lymph node (LN) stage, Tumor Grade, RS score and Chemotherapy were selected for OS nomogram, while Age, Tumor size, LN stage, Tumor Grade, RS score, Surgery were selected for BCSS nomogram. The hazard ratios for selected variable were summarized in Table 3.

**Table 3:** Selected variables for OS and BCSS according to Cox regression model.

Variable	OS		BCSS	
	HR (95%CI)	P	HR (95%CI)	P
Age	1.07 (1.06–1.07)	<0.001	1.03 (1.02–1.04)	<0.001
Tumor size	1.01 (1.01–1.02)	<0.001	1.01 (1.00–1.02)	<0.001
LN stage		<0.001		<0.001
0	Ref		Ref	
1	1.46 (1.27–1.68)		1.48 (1.26–1.76)	
2	2.22 (1.43–3.44)		3.00 (1.84–4.89)	
3	4.91 (2.86–8.42)		6.52 (3.55–11.96)	
Grade		0.001		<0.001
1	Ref		Ref	
2	1.06 (0.92–1.21)		1.29 (1.07–1.54)	
3	1.34 (1.13–1.60)		1.58 (1.26–1.97)	
RS score	1.03 (1.03–1.04)	<0.001	1.04 (1.03–1.05)	<0.001
Chemotherapy		0.03	–	–
No	Ref		–	–
Yes	0.85 (0.73–0.99)		–	–
Surgery	–	–		0.014
No	–	–	Ref	
Yes	–	–	0.30 (0.10–0.94)	

**Fig. 4:**
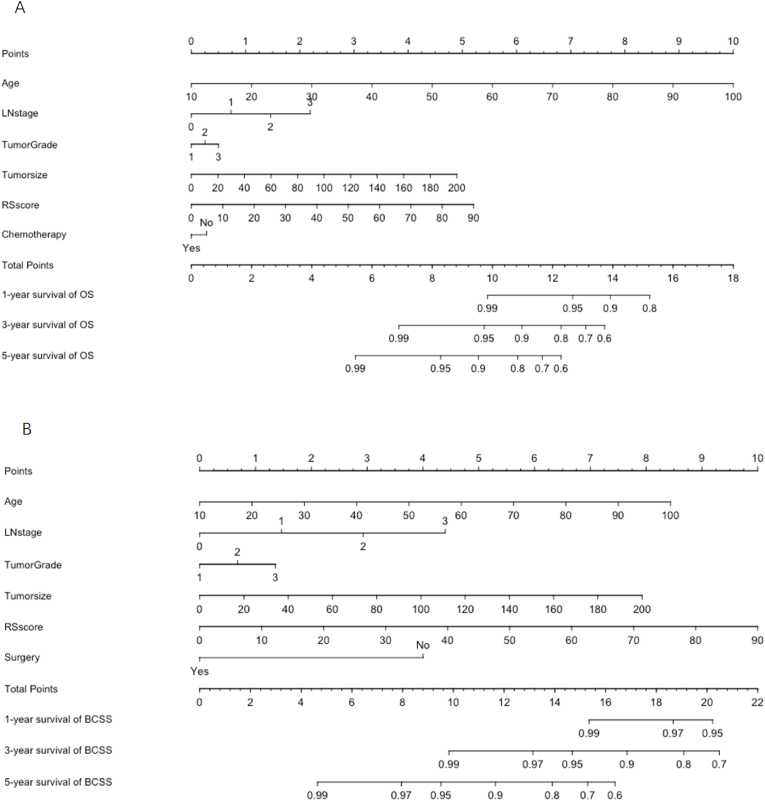
Nomogram predicting 1-year、3-year and 5-year survival of OS and BCSS for BC patients combined CTS5 score and RS score.

**Fig. 5:**
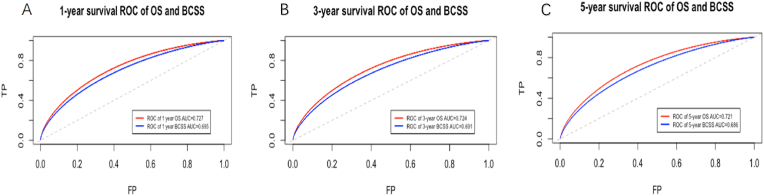
Survival ROC of OS and BCSS according to nomogram model for patients with different follow-up months.

**Fig. 6:**
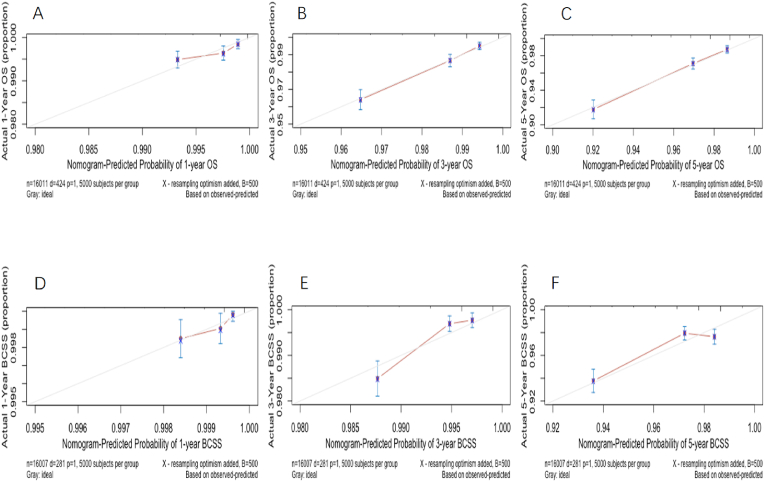
Calibration curve of OS and BCSS according to nomogram model for patients with different survival months.

Nomogram predicting 1-year,3-year and 5-year survival of OS and BCSS were shown in Fig. 4. In training dataset, the C-index for OS and BCSS nomogram were 0.72 (95%CI 0.71–0.74) and 0.70 (95%CI 0.68–0.71), respectively. In validation dataset, the corresponding C-index for OS and BCSS were 0.72 (95%CI 0.69–0.75) and 0.71 (95%CI 0.67–0.74), respectively. The survival ROC curve of nomogram for OS and BCSS in validation dataset were shown in Fig. 5. The AUC of OS and BCSS nomogram were significantly higher than either CTS5 or RS risk alone (*P*＜0.01). Fig. 6 showed the calibration plot of OS and BCSS nomograms.

**Fig. 7:**
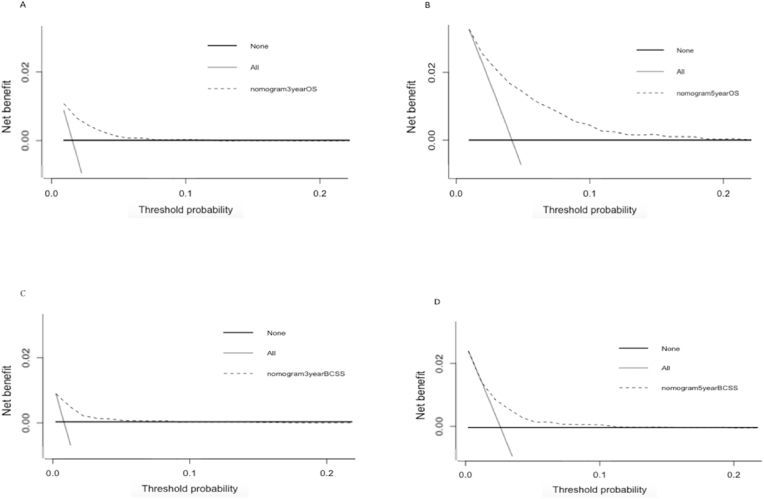
DCA curve of OS and BCSS according to nomogram model for patients with different follow-up months.

The calibration plots indicated the predicted values generated by the novel nomograms had a good consistency with the observed values. For clinical utility, Fig. 7 exhibited the decision curve analysis (DCA) of nomogram, indicating the nomogram had potential for clinical utility. The DCA indicated that when the threshold probability for 3-year and 5-years OS were within the range 2–7% and 2%–18%, the nomogram added more net benefit than “all or none” strategy. And the nomogram was both effective for BCSS and OS prediction.

## 4 Discussion

The present study analyzed large-scale population with 64044 breast cancer patients to investigate the performance of CTS5 and RS model, and develop a novel nomogram for prognosis prediction. For model discrimination, CTS5 model was superior to RS for OS prediction. RS model showed better performance for BCSS in late recurrence subsets and worse performance in early and intermediate recurrence subsets than CTS5. For calibration, CTS5 model was superior to RS model for OS, which overestimated the recurrence risk in low-risk subgroup. Both models overestimated the risk for BCSS. The two models had no significant difference in terms of NRI. And the nomogram which combined CTS5 and RS improved the discrimination and calibration power.

In the era of precision medicine, the clinicians increasingly rely on tumor molecular analysis to predict recurrence risk and tailor adjuvant treatment. Both the American Society of Clinical Oncology and National Comprehensive Cancer Network guidelines recommend Oncotype DX for adjuvant chemotherapy decision-making. The NSABP-B14 study prospectively validated the RS as a predictor of recurrence, irrespective of age and tumor size [4]. The NSABP B-20 study also proved that the RS can predict recurrence of node-negative breast cancer patients [5]. Previous study showed that the RS altered recommendations for adjuvant chemotherapy in node-positive patients as well [11]. The NSABP-B28 study further demonstrated that RS was an independent predictor for disease-free and overall survival. It was concordant with the result of the present study. Study by Paik et al. suggested RS was also useful for predicting late recurrence (>10 years) [4]. Whereas, other studies reached contradictory conclusion [12,13]. Our study also showed RS risk model was prognostic for 1-year, 3-year and 5-year survival of BCSS and OS, but compared with CTS5, there was no significant difference for model performance in terms of either discrimination or calibration. Since CTS5 risk model was based on clinicopathological variables with high data availability and low cost, it may serve as a reasonable alternative for genomic assays such as RS. Additionally, RS have several limitations. It was unable to reflect tumor heterogeneity and manage multifocal breast cancer [14]. And theoretically, RS contained merely genetic information and did not cover important clinicopathological parameters, such as age and tumor grade. TAILORx study revealed that patients with RS intermediate risk remained undetermined for chemotherapy administration and suggested that traditional prognostic factors should be considered to facilitate decision making [7].

Overestimation remained one of the major concerns for clinical application of CTS5 and RS model. The calibration of CTS5 for late recurrence was still controversial and it may overestimate the recurrence rate of HR + breast cancer. Study by Lee et al. found that the actual incidence of late DR was low in the low-risk CTS5 score group [3]. Our study evaluated the prognostic value of CTS5 in large-scale population and drew similar conclusion. It was concordant with several previous studies that CTS5 model could predict the prognosis of BC patients [3,15,16]. In a combined analysis of the TEAM and IDEAL trials, CTS5 overestimated late distant recurrence risk [17]. Our study also suggested CTS5 risk model gave higher prediction value than observation for 3-year and 5-year recurrence risk estimation for both BCSS and OS. Similarly, RS model also had the same trend with overestimation for both OS and BCSS, but it had larger deviation between prediction and observation for OS than CTS5.

In the present study, we further evaluated the correlation between CTS5 performance and cancer recurrence timing. CTS5 model was prognostic not only for 5-year long-term survival, but also short-term survival (1-year and 3-year survival). It implied the traditional clinicopathological parameters included in CTS5 model had a long-lasting impact (>5 years) on patient survival and CTS5 could be a useful prognostic model both for early and late recurrence. Although RS mainly used for prediction of early recurrence, it presented better discrimination for BCSS in late recurrence subset. Regarding menopausal status, CTS5 model was developed from a large postmenopausal HR + breast cancer cohort and served as an effective prediction tool for evaluating long-term recurrence risk. Richman et al. revealed that calibration of the CTS5 was even better in patients who did not receive extended endocrine therapy [15].Although CTS5 model was derived from postmenopausal patients, our conclusion supported the notion that its application could be extrapolated to premenopausal women. Similarly, several studies proved that CTS5 was effective both in postmenopausal and premenopausal women [3,16,18]. And in our previous work, CTS5 model was also proved that its prognostic value was persistent in HR+/HER2-negetive patients irrespective of menopausal status [16]. These studies supported the use of CTS5 model for estimating the risk of late recurrence in premenopausal women.

Furthermore, although CTS5 and RS had comparable prognostic value, they were based on different parameters and had no overlaps. CTS5 was calculated using purely clinicopathological data, while RS was based on genomic information only. Hence, theoretically, combined CTS5 and RS may be helpful to develop more accurate risk model. Besides, several studies also reported that the association between age and RS prognostic value, indicating the potential relevance between clinicopathological and genomic data [19]. The present study combined age, RS, CTS5 score and treatment information to develop and validate a novel nomogram to predict breast cancer survival. Compared to either CTS5 or RS model alone, the nomogram exhibited an improved performance for survival prediction with both better discrimination and calibration. The results of DCA also demonstrated that the nomogram had a strong potential for clinical usage. In addition, the nomogram model could bring convenience for both clinicians and patients. Similarly, several risk models were also developed in this clinical-genetic manner. The EPclin score is a prediction model which combined the EP (EndoPredict) gene test with nodal status and tumor size. It was developed based on retrospective analysis of a combined cohort from the ABCSG-6 and ABCSG-8 phase III trials, and could predict favorable prognosis in patients with intermediate or high risk [20]. Sparano et al. has developed a new online tool called “RSClin” with combination of genomic and clinical parameters to predict distant recurrence. It derived from B-14 and TAILORx trials and had more prognostic power for DR than RS or clinical-pathological factors alone [21].

Our study also has several limitations. First, the present study was based on data from SEER database and was a retrospective study. The above findings should be validated in large prospective studies. Second, given this portion of patients with RS data did not include the menopausal status and HER2 receptor status in the SEER database, these two factors may affect the evaluation of model performance. Third, there was no accurate treatment information provided in SEER database, and different treatment may affect the prognosis.

## 5 Conclusion

In conclusion, our study proved the CTS5 risk model had comparable prognostic value as RS in HR + breast cancer patients. And the nomogram which combined the CTS5 and RS had a better performance than both CTS5 and RS in terms of OS and BCSS prediction. This novel nomogram warrants future large-scale randomized controlled study to further validate its prognostic value.

## Ethics approval

Not applicable.

## Data availability statement

The datasets used or analyzed during the current study are available from the corresponding author on reasonable request.

## Author contributions

Conception and design: Qiang Sun, Ying Xu and Changjun Wang. Data analysis: Ying Xu and Changjun Wang and Yan Lin. Manuscript draft written: Ying Xu and Changjun Wang. Manuscript revision: Yidong Zhou, Feng Mao, Xiaohui Zhang, Songjie Shen, Yanna Zhang, Qiang Sun.

## Funding

Key Projects in the National Science & Technology Pillar Program during the Twelfth Five-year Plan Period (2014BAI08B00). 10.13039/501100011408State Key Laboratory of Medicinal Chemical Biology (NanKai University) (No. 2019014)

## Declaration of competing interest

The authors declare that they have no known competing financial interests or personal relationships that could have appeared to influence the work reported in this paper.
