# Supplementary material for: Comparison of CTS5 risk model and 21-gene recurrence score assay in large-scale breast cancer population and combination of CTS5 and recurrence score to develop a novel nomogram for prognosis prediction
Source: Breast. 2022 Mar 16;63:61–70. doi: 10.1016/j.breast.2022.03.007 (PMC8942860; doi:10.1016/j.breast.2022.03.007)
Supplement: Multimedia component 1 [file mmc1.docx]

**Supplementary material**

| Model | | Early recurrence subset | | | | Intermediate recurrence subset | | | Late recurrence subset | | | | |
| --- | --- | --- | --- | --- | --- | --- | --- | --- | --- | --- | --- | --- | --- |
|  |  | OS | | BCSS | | OS | | BCSS | | OS | | BCSS | |
|  |  | HR (95%CI) | P value | HR (95%CI) | P value | HR (95%CI) | P value | HR (95%CI) | P value | HR (95%CI) | P value | HR (95%CI) | P value |
| CTS5 | Low-risk | 1 | 1 | 1 | 1 | 1 | 1 | 1 | 1 | 1 | 1 | 1 | 1 |
|  | Intermediate-risk | 1.94(1.67-2.67) | <0.001 | 2.99(2.24-4.00) | <0.001 | 2.51(2.10-3.01) | <0.001 | 1.64(1.40-1.91) | <0.001 | 1.88(1.40-2.52) | <0.001 | 2.03(1.23-3.32) | 0.005 |
|  | High-risk | 3.89(3.28-4.62) | <0.001 | 7.96(5.91-10.72) | <0.001 | 4.22(3.39-5.25) | <0.001 | 2.38(1.94-2.92) | <0.001 | 4.83(3.48-6.72) | <0.001 | 4.66(2.63-8.25) | <0.001 |
| RS | Low-risk | 1 | 1 | 1 | 1 | 1 | 1 | 1 | 1 | 1 | 1 | 1 | 1 |
|  | Intermediate-risk | 1.22(1.05-1.41) | <0.001 | 2.53(1.90-3.37) | <0.001 | 1.30(1.09-1.54) | <0.001 | 1.26(1.08-1.47) | <0.001 | 1.22(0.92-1.62) | 0.164 | 2.47(1.47-4.15) | <0.001 |
|  | High-risk | 2.72(2.25-3.30) | <0.001 | 9.02(6.63-12.26) | <0.001 | 2.69(2.13-3.40) | <0.001 | 2.18(1.75-2.71) | <0.001 | 2.85(1.98-4.10) | <0.001 | 7.01(3.88-12.67) | <0.001 |

Table S1 Survival analyses for OS and BCSS in different recurrence risk subset according to CTS5 risk model and RS risk model

Table S2 Net reclassification index (NRI) of OS according to CTS5 risk model and RS risk model

1. follow-up time <36 months

| Patients without events(n=63176) | | **RS model** | | | |
| --- | --- | --- | --- | --- | --- |
|  |  | Low risk | Moderate risk | High risk | Total-CTS5 |
| **CTS5 model** | Low risk | 25312 | 12841 | 1575 | 39728 |
|  | Moderate risk | 9327 | 6469 | 1776 | 17572 |
|  | High risk | 2831 | 2136 | 909 | 5876 |
|  | Total-RS | 37470 | 21446 | 4260 | 63176 |

| Patients with events(n=868) | | **RS model** | | | |
| --- | --- | --- | --- | --- | --- |
|  |  | Low risk | Moderate risk | High risk | Total-CTS5 |
| **CTS5 model** | Low risk | 215 | 120 | 21 | 356 |
|  | Moderate risk | 137 | 116 | 54 | 307 |
|  | High risk | 75 | 68 | 62 | 205 |
|  | Total-RS | 427 | 304 | 137 | 868 |

|  | Patients with event (No.) | Patients without event (No.) |  |
| --- | --- | --- | --- |
| Correct reclassification | 195 | 14294 |  |
| Incorrect reclassification | 280 | 16192 |  |
| Net reclassification | -85 | -1898 |  |
| NRI=-0.03(95%CI -0.036-0.319) Bootstrap=10000 | | | |

1. follow-up time 36-60 months

| Patients without events(n=36338) | | **RS model** | | | |
| --- | --- | --- | --- | --- | --- |
|  |  | Low risk | Moderate risk | High risk | Total-CTS5 |
| **CTS5 model** | Low risk | 14514 | 7751 | 930 | 23195 |
|  | Moderate risk | 5227 | 3727 | 1036 | 9990 |
|  | High risk | 1509 | 1154 | 490 | 3153 |
|  | Total-RS | 21250 | 12632 | 2456 | 36338 |

| Patients with events(n=540) | | **RS model** | | | |
| --- | --- | --- | --- | --- | --- |
|  |  | Low risk | Moderate risk | High risk | Total-CTS5 |
| **CTS5 model** | Low risk | 115 | 68 | 20 | 203 |
|  | Moderate risk | 90 | 90 | 39 | 219 |
|  | High risk | 45 | 47 | 26 | 118 |
|  | Total-RS | 250 | 205 | 85 | 540 |

|  | Patients with event (No.) | Patients without event (No.) |  |
| --- | --- | --- | --- |
| Correct reclassification | 127 | 7890 |  |
| Incorrect reclassification | 182 | 9717 |  |
| Net reclassification | -55 | -1827 |  |
| NRI=-0.182(95%CI -0.253-0.115) Bootstrap=10000 | | | |

1. follow-up time ≥60 months

| Patients without events(n=15322) | | **RS model** | | | |
| --- | --- | --- | --- | --- | --- |
|  |  | Low risk | Moderate risk | High risk | Total-CTS5 |
| **CTS5 model** | Low risk | 6173 | 3677 | 461 | 10311 |
|  | Moderate risk | 2205 | 1170 | 476 | 3851 |
|  | High risk | 540 | 430 | 190 | 1160 |
|  | Total-RS | 8918 | 12632 | 1127 | 15322 |

| Patients with events(n=180) | | **RS model** | | | |
| --- | --- | --- | --- | --- | --- |
|  |  | Low risk | Moderate risk | High risk | Total-CTS5 |
| **CTS5 model** | Low risk | 37 | 28 | 11 | 76 |
|  | Moderate risk | 23 | 23 | 8 | 54 |
|  | High risk | 24 | 17 | 9 | 50 |
|  | Total-RS | 84 | 68 | 28 | 180 |

|  | Patients with event (No.) | Patients without event (No.) |  |
| --- | --- | --- | --- |
| Correct reclassification | 47 | 3175 |  |
| Incorrect reclassification | 64 | 4614 |  |
| Net reclassification | -17 | -1439 |  |
| NRI=-0.181(95%CI -0.332-0.063) Bootstrap=10000 | | | |

Table S3 Net reclassification index (NRI) of BCSS according to CTS5 risk model and RS risk model

1. follow-up time <36 months

| Patients without events(n=63764) | | **RS model** | | | |
| --- | --- | --- | --- | --- | --- |
|  |  | Low risk | Moderate risk | High risk | Total-CTS5 |
| **CTS5 model** | Low risk | 25493 | 12925 | 1586 | 40004 |
|  | Moderate risk | 9445 | 6529 | 1799 | 17773 |
|  | High risk | 2880 | 2179 | 928 | 5987 |
|  | Total-RS | 37818 | 21633 | 4313 | 63764 |

| Patients with events(n=280) | | **RS model** | | | |
| --- | --- | --- | --- | --- | --- |
|  |  | Low risk | Moderate risk | High risk | Total-CTS5 |
| **CTS5 model** | Low risk | 34 | 36 | 10 | 80 |
|  | Moderate risk | 19 | 56 | 31 | 106 |
|  | High risk | 26 | 25 | 43 | 94 |
|  | Total-RS | 79 | 117 | 84 | 280 |

|  | Patients with event (No.) | Patients without event (No.) |  |
| --- | --- | --- | --- |
| Correct reclassification | 77 | 14504 |  |
| Incorrect reclassification | 70 | 16310 |  |
| Net reclassification | 7 | -1806 |  |
| NRI=-0.011(95%CI -0.128-0.229) Bootstrap=10000 | | | |

1. follow-up time 36-60 months

| Patients without events(n=36161) | | **RS model** | | | |
| --- | --- | --- | --- | --- | --- |
|  |  | Low risk | Moderate risk | High risk | Total-CTS5 |
| **CTS5 model** | Low risk | 14418 | 7693 | 924 | 23035 |
|  | Moderate risk | 5212 | 3722 | 1032 | 9966 |
|  | High risk | 1512 | 1154 | 494 | 3160 |
|  | Total-RS | 21142 | 12569 | 2450 | 36161 |

| Patients with events(n=717) | | **RS model** | | | |
| --- | --- | --- | --- | --- | --- |
|  |  | Low risk | Moderate risk | High risk | Total-CTS5 |
| **CTS5 model** | Low risk | 211 | 126 | 26 | 363 |
|  | Moderate risk | 105 | 95 | 43 | 243 |
|  | High risk | 42 | 47 | 22 | 111 |
|  | Total-RS | 358 | 268 | 91 | 717 |

|  | Patients with event (No.) | Patients without event (No.) |  |
| --- | --- | --- | --- |
| Correct reclassification | 195 | 7878 |  |
| Incorrect reclassification | 194 | 9649 |  |
| Net reclassification | 1 | -1771 |  |
| NRI=-0.07(95%CI -0.139-0.009) Bootstrap=10000 | | | |

1. follow-up time ≥60 months

| Patients without events(n=15806) | | **RS model** | | | |
| --- | --- | --- | --- | --- | --- |
|  |  | Low risk | Moderate risk | High risk | Total-CTS5 |
| **CTS5 model** | Low risk | 6201 | 3693 | 465 | 10359 |
|  | Moderate risk | 2220 | 1607 | 480 | 4307 |
|  | High risk | 561 | 438 | 195 | 1194 |
|  | Total-RS | 8982 | 5738 | 1140 | 15860 |

| Patients with events(n=66) | | **RS model** | | | |
| --- | --- | --- | --- | --- | --- |
|  |  | Low risk | Moderate risk | High risk | Total-CTS5 |
| **CTS5 model** | Low risk | 9 | 12 | 7 | 28 |
|  | Moderate risk | 8 | 10 | 4 | 22 |
|  | High risk | 3 | 9 | 4 | 16 |
|  | Total-RS | 20 | 31 | 15 | 66 |

|  | Patients with event (No.) | Patients without event (No.) |  |
| --- | --- | --- | --- |
| Correct reclassification | 23 | 3219 |  |
| Incorrect reclassification | 20 | 4638 |  |
| Net reclassification | 3 | -1419 |  |
| NRI=-0.048(95%CI -0.268-0.205) Bootstrap=10000 | | | |

Table S4 Demographic and Clinical Characteristics of Patients in training and validation dataset

| Characteristic | Training dataset  N=48033（%） | Validation dataset  N=16011（%） | *P* value |
| --- | --- | --- | --- |
| Age |  |  | 0.275* |
| Mean±sd | 58.23±10.55 | 58.45±10.56 |  |
| ≤45 | 4908(10.2) | 1586(9.） |  |
| 45-65 | 28523(59.4) | 9469(59.1) |  |
| ＞65 | 14602(30.4) | 4956(30.9) |  |
| Race |  |  | 0.757 |
| White | 39256(81.7) | 13034(81.4) |  |
| Black | 3944(8.2) | 1364(8.5) |  |
| Asian | 4321(9.0) | 1440(9.0) |  |
| Other | 214(0.5) | 68(0.4) |  |
| Unknown | 298(0.6) | 105(0.7) |  |
| Tumor size（mm） |  |  | 0.275* |
| ≤20 | 35024(72.9) | 11603(72.5) |  |
| 20-50 | 12108(25.2) | 4116(25.7） |  |
| ＞50 | 901(1.9) | 292(1.8） |  |
| Grade |  |  | 0.127* |
| I | 13949(29.0) | 4592(28.7) |  |
| II | 26424(55.0） | 8827(55.1） |  |
| III | 7660(16.0） | 2592(16.2） |  |
| LN（+） |  |  | 0.563* |
| 0 | 39945(83.2) | 13296(83.0) |  |
| 1-3 | 7731(16.0) | 2588(16.2) |  |
| 4-9 | 279(0.6) | 96(0.6) |  |
| ＞9 | 78(0.2) | 31(0.2) |  |
| ER |  |  | 0.611 |
| Negative | 45(0.1) | 18(0.1) |  |
| Positive | 47988(99.9) | 15993(99.9) |  |
| PR |  |  | 0.285 |
| Negative | 4068(8.5) | 1312(8.2) |  |
| Positive | 43965(91.5） | 14699(91.8) |  |
| HER2 |  |  | 1 |
| Negative | 47120(98.1) | 15706(98.1) |  |
| Positive | 913(1.9) | 305(1.9) |  |
| Subtype |  |  | 1 |
| Luminal A | 47120(98.1) | 15706(98.1) |  |
| Luminal B | 913(1.9） | 305(1.9） |  |
| TNM Stage |  |  | 0.387* |
| I | 32169(67.0) | 10685(66.7) |  |
| II | 15112(31.5） | 506(31.6） |  |
| III | 685(1.4) | 231(1.4) |  |
| IV | 67(0.1) | 31(0.2) |  |
| Surgery |  |  | 0.099 |
| No | 55(0.1) | 10(0.06) |  |
| Yes | 47964(99.86） | 15997(99.91) |  |
| Unknown | 14(0.03) | 4(0.03) |  |
| Radiotherapy |  |  | 0.669 |
| No | 17477(36.4) | 5804(36.3) |  |
| Yes | 29387(61.2) | 9841(61.5) |  |
| Unknown | 1169(2.4) | 366(2.3) |  |
| Chemotherapy |  |  | 0.662 |
| No | 37981(79.1) | 12687(79.2) |  |
| Yes | 10052(20.9) | 3324(20.8) |  |
| RS risk |  |  | 0.275* |
| low | 28447(59.2) | 9450(59.1) |  |
| Intermediate | 16301(33.9) | 5449(34.0) |  |
| High | 3285(6.9) | 1112(6.9) |  |
| CTS5 risk |  |  | 0.127* |
| low | 30108(62.7) | 9975(62.3) |  |
| Intermediate | 13408(27.9) | 4474(27.9) |  |
| High | 4517(9.4) | 1562(9.8) |  |

P value of Person’s Chi-square test; * P value of Kruskal-Wills test


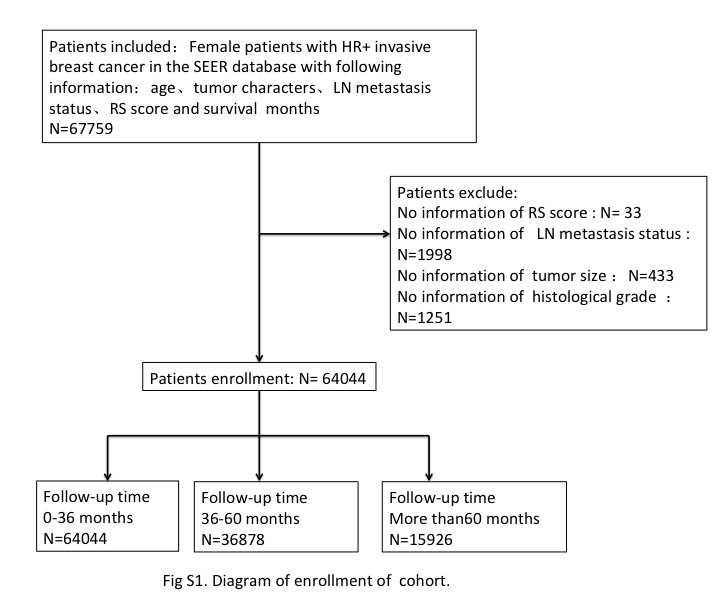


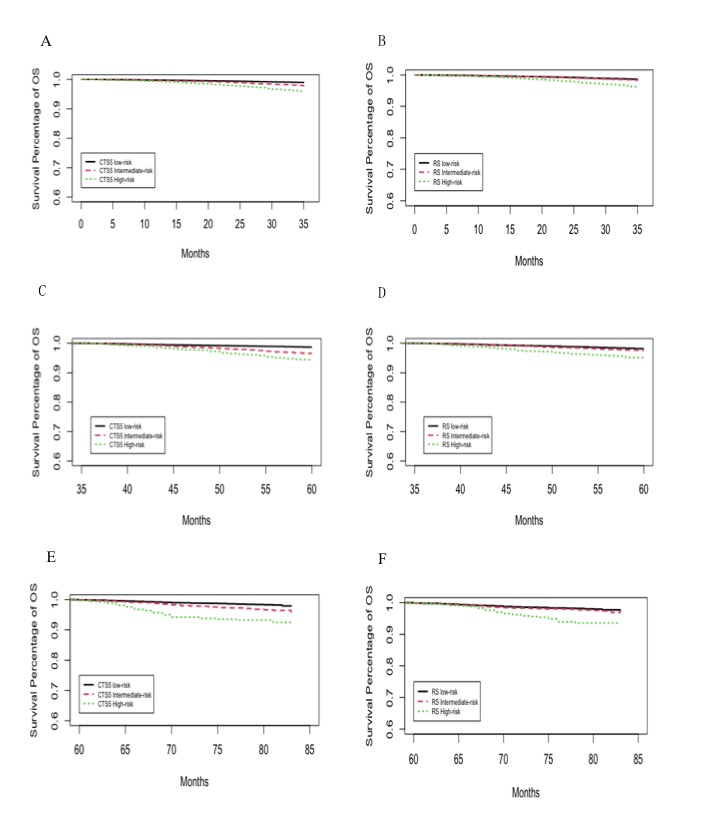


Fig S2. Kaplan-Meier curves of OS according to CTS5 risk model and RS risk model for patients with different follow-up months.


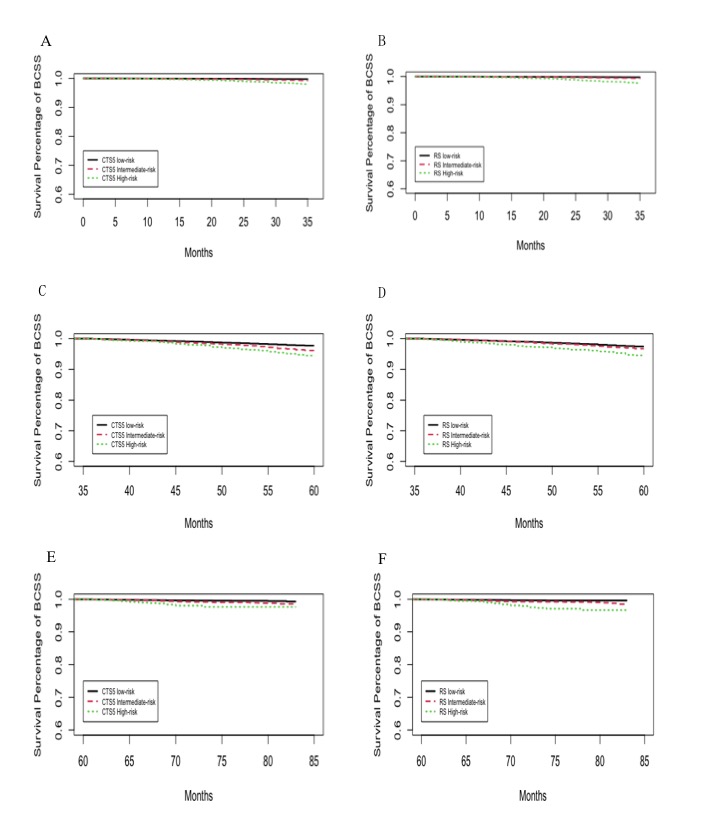


Fig S3. Kaplan-Meier curves of BCSS according to CTS5 risk model and RS risk model for patients with different follow-up months.
